# Image-Guided Ablation for Colorectal Liver Metastasis: Principles, Current Evidence, and the Path Forward

**DOI:** 10.3390/cancers13163926

**Published:** 2021-08-04

**Authors:** Yuan-Mao Lin, Iwan Paolucci, Kristy K. Brock, Bruno C. Odisio

**Affiliations:** 1 Department of Interventional Radiology, Division of Diagnostic Imaging, The University of Texas MD Anderson Cancer Center, Houston, TX 77030, USA; ylin22@mdanderson.org (Y.-M.L.); IPaolucci@mdanderson.org (I.P.); 2 Department of Imaging Physics, Division of Diagnostic Imaging, The University of Texas MD Anderson Cancer Center, Houston, TX 77030, USA; kkbrock@mdanderson.org

**Keywords:** colorectal liver metastasis, radiofrequency, irreversible electroporation, microwave, ablation, cryoablation, survival, local tumor progression

## Abstract

**Simple Summary:**

Colorectal cancer is the fourth most common type of cancer globally. Approximately 20% of patients with colorectal cancer present with synchronous liver metastases, and up to 60% will develop metachronous metastases during the course of the disease. Although liver resection is currently considered the local treatment of choice for colorectal liver metastasis (CLM), less than one-third of patients are eligible for surgery at the time of diagnosis of CLM. Ablation is a well-established, less invasive, locoregional therapy for patients with small CLMs, which has shown favorable oncological outcomes in patients with unresectable CLMs, comparable to those in patients eligible for surgery. The increasing knowledge of factors affecting oncological outcomes has allowed selected patients with resectable small volume CLMs to be treated with thermal ablation with curative intent. The continuous technological evolutions in imaging and image guidance have contributed to this paradigm shift in CLM treatment. The importance of patient selection, patient factors, tumor factors, ablation techniques, and clinical applications is discussed in this article.

**Abstract:**

Image-guided ablation can provide effective local tumor control in selected patients with CLM. A randomized controlled trial suggested that radiofrequency ablation combined with systemic chemotherapy resulted in a survival benefit for patients with unresectable CLM, compared to systemic chemotherapy alone. For small tumors, ablation with adequate margins can be considered as an alternative to resection. The improvement of ablation technologies can allow the treatment of tumors close to major vascular structures or bile ducts, on which the applicability of thermal ablation modalities is challenging. Several factors affect the outcomes of ablation, including but not limited to tumor size, number, location, minimal ablation margin, *RAS* mutation status, prior hepatectomy, and extrahepatic disease. Further understanding of the impact of tumor biology and advanced imaging guidance on overall patient outcomes might help to tailor its application, and improve outcomes of image-guided ablation.

## 1. Introduction

Colorectal cancer is the fourth most common malignancy and the third leading cause of cancer-related death in the world [1]. Approximately 20% of patients with colorectal cancer present with synchronous liver metastases, and up to 60% will develop metachronous metastases during the course of the disease [2]. The liver is the most common site of metastases, and liver metastases are negatively associated with overall survival [2]. Liver resection is currently considered the local treatment of choice for colorectal liver metastasis (CLM), with reported 5-year overall survival rates of up to 58% in selected patients [3]. However, only 20–30% of patients are eligible for resection, based on the patient’s medical status and the extent of the disease [4]. Ablation is a well-established locoregional therapy for patients with small CLMs. Additionally, ablation has shown encouraging results for potentially resectable CLMs [5,6,7,8]. An ongoing randomized study is investigating the use of ablation as a first-line local approach for the treatment of CLM [9]. The role of ablation is increasing, and the treatment paradigm of CLM patients is evolving. This article aims to review the role of ablation, as well as to discuss the future directions of this treatment modality on the management of patients with CLM.

## 2. Patient Selection

The introduction of novel target agents along with evolving chemotherapy regimens has been linked with significant improvements in the outcomes of patients with CLM, as reflected in increased median survival up to 31 months in selected patients with unresectable CLM [10,11,12,13], as well with the conversion of CLM patients deemed unresectable to resectable. Despite such advances, systemic chemotherapy is considered a palliative treatment, unless the patients can undergo resection or ablation and their derived benefits [14]. In this context, liver ablation has been utilized in a wide variety of clinical presentations, ultimately aiming to provide a local curative-intent option for patients with liver-only or liver-dominant colorectal metastasis.

Commonly, ablation is applied for patients who present with small and limited CLMs. Image-guided ablation is particularly effective in treating small- to medium-sized tumors. The most commonly used cutoff maximal diameter is 3 cm, as a tumor size up to 3 cm is an independent predictor for oncological outcomes that can be compared to outcomes of surgical resection [15,16,17]. For tumors measuring up to 5 cm, complete tumor eradication can be achieved, depending on proper anatomical location and multiple overlapping ablations [7,18]. Image-guided ablation is generally not recommended for tumors larger than 5 cm, given high rates of local tumor progression (LTP) [7,19]. Universally, a small solitary CLM is ideal for image-guided ablation, with 5-year overall survival rates up to 51% [14,20]. However, patients with up to five tumors are also eligible for image-guided ablation [14]. For tumor size ≤ 3 cm, number ≤ 3, and no extrahepatic disease, image-guided ablation with ablation margins > 10 mm has been shown to offer similar local tumor control to hepatectomy alone [21]. The use of ablation on patients with liver-only resectable CLMs up to 3 cm varies among institutions; some reserve it for patients who refused surgery, while others apply ablation as the first local modality—along with the “test-of-time” concept—with the aim of treating the disease while limiting the number of liver resections performed on patients who might ultimately develop additional metastases [22]. Ablation may even become the preferred go-to local treatment, depending on the results of a phase III randomized controlled trial comparing liver resection to thermal ablation for patients presenting with CLMs up to 3 cm, with expected results in 2025 [9,23].

Tumor location can affect both local tumor control and complications. Care should be taken when performing ablation on tumors located next to central bile ducts, vessels, or other vulnerable structures that cannot be protected with hydrodissection or other protective techniques. The use of thermal ablation to treat tumors adjacent to the central bile ducts has been associated with increased risk of bile duct injury and subsequent complications, such as cholangitis or liver abscess. It has been reported that patients with prior use of intrahepatic chemotherapy or bevacizumab, as well with pre-existing biliary dilatation, are at higher risk of developing biliary complications [24,25]. Thermal ablation close to large vessels increases the risk of residual tumor and early LTP due to the heat-sink phenomenon, as well as venous thrombosis and, consequently, liver infarction [26,27,28,29]. In such situations, non-thermal ablation techniques such as irreversible electroporation (IRE) can be applied [30,31]. Additionally, more intensive ablation strategies or the use of microwave ablation (MWA) should be considered to compensate for the heat-sink phenomenon. Studies have demonstrated that MWA is effective on perivascular tumors with satisfactory ablation margins [21,32]. Although the optimal patient for ablation is one with disease limited to the liver, ablation can benefit patients with limited extrahepatic diseases [20,33,34]. Palliative liver ablation in patients with extensive extrahepatic metastases is not currently recommended.

Image-guided ablation is recommended as a safe and effective locoregional therapy for CLM patients [15,35,36,37]. Ablation combined with systemic chemotherapy has been shown in a prospective randomized phase II study to significantly prolong the overall survival in patients with unresectable CLMs when compared to the use of chemotherapy alone [38]. Hepatectomy combined with intraoperative or postoperative ablation can achieve local tumor control and preserve the remaining liver in patients who have limited liver reserves, such as those with extended liver distribution or previous major liver resection [39,40,41]. Furthermore, postoperative ablation can be used to treat CLMs that were not able to be identified during intraoperative ultrasound evaluation. In such circumstances, cross-sectional (CT or MR) guided ablation has been shown to be an effective and safe approach [42]. For recurrent and new tumors, it is preferable to use image-guided ablation to treat them repeatedly, while limiting the destruction of the liver parenchyma and providing survival rates similar to patients without recurrence [43,44,45,46]. Moreover, image-guided ablation is considered to be the treatment of choice in patients with technically resectable disease who cannot undergo surgery due to medical comorbidities. Several publications have reported that ablation leads to lower post-treatment morbidity, lower complication rates, and shorter hospital stays. Repeat ablation can also be utilized as a “test-of-time” strategy, in order to reduce unnecessary metastasectomy on patients with poor tumor biology [22,47]. This so-called “test-of-time” strategy offers thermal ablation ahead of liver resection, and is advocated for patients with small tumors in order to spare surgically eligible patients who would develop extended liver disease from potentially unnecessary liver resection or remain disease-free after ablation [22,47]. Those with local failure who remain candidates for local therapy can still be treated by hepatectomy if and when ablation fails locally [22,47].

## 3. Technique

### 3.1. Ablation Modalities

Radiofrequency ablation (RFA) and MWA ablations are thermal ablation modalities that are widely used as the standard of care to treat CLM. Moreover, IRE has been applied for patients who are not amenable to thermal ablation due to the higher risk of thermal injury to vital structures—such as central bile ducts—in proximity to the ablation zone.

#### 3.1.1. Radiofrequency Ablation

This widely available technique uses an interstitial electrode to produce an alternating electric current to the target tissue. The electric current oscillates tissue ions rapidly and creates frictional heating. When the temperatures of the target tissue are between 60 °C and 100 °C, protein denaturation, coagulative necrosis, and immediate cell death occur. The electrical conductance is degraded above 100 °C due to the water vaporization and tissue carbonization, limiting the amount of energy that can be delivered to eradicate the tumor, and resulting in a suboptimal treatment effect. A major limitation of RFA is the heat-sink effect that occurs if the target lesion abuts a blood vessel larger than 3 mm. Another disadvantage is the thermal injury to vital structures adjacent to the ablated area, such as bile ducts and vessels. For this reason, applying RFA to tumors close to central bile ducts and vessels is challenging, and sometimes contraindicated, because of suboptimal local tumor control and higher rates of complications [48,49].

#### 3.1.2. Microwave Ablation

This technique creates an electromagnetic spectrum with frequencies from 900 to 2450 MHz that generate heat by agitating surrounding water molecules. Theoretically, MWA generates greater heat and less heat-sink effect than RFA, creating larger ablation zones in a shorter period. Studies have reported that MWA is more effective than RFA for perivascular tumors [21,32,50,51]. For peribiliary tumors, the complication rates are higher for MWA than for RFA (57 vs. 3%; *p* = 0.002) [50]. Retrospective studies have reported that MWA leads to lower rates of LTP than RFA (MWA: 6–10% vs. RFA: 20–20.3%) and a lower 2-year cumulative local recurrence rate (7 vs. 18%; *p* = 0.01) [52,53,54]. However, another study reported no difference in the cumulative local tumor recurrence rates between MWA and RFA after stratifying outcomes by ablation margin size [21]. Regarding overall survival and ablation-related complications, there was no significant difference between MWA and RFA in the above literature [21,52,53,54]. For treating primary and metastatic liver tumors, a meta-analysis comparing MWA and RFA reported no significant differences in overall survival, disease-free survival, local recurrence rates, or adverse events [55]. There are no randomized studies comparing MWA to RFA, and it may be that the two techniques have complementary rather than competing roles.

#### 3.1.3. Cryoablation

Cryoablation uses extreme cold generated by the Joule–Thomson effect to freeze and destroy tumors. The features of cryoablation are the repetition of the freeze–thaw cycles. The rapid freezing first forms extracellular ice, causing a hyperosmotic state that draws the intracellular free water and causes damage to the cell; as temperatures drop, intracellular ice forms, causing further damage. This is followed by a slow thaw cycle, in which ice melts and water enters the cell, causing expansion and disruption to the cell. Although cryoablation was one of the earliest ablation techniques used to treat CLM, it has been associated with higher rates of local recurrence and complications [56], and is therefore rarely applied in clinical practice. A study of 212 patients including 77 patients with CLM treated by percutaneous cryoablation showed the 3-year LTP rate was 21.6%, and the 5-year overall survival rate was around 20% for CLM [57]. The overall complication rate was 5.8% in the whole cohort of 212 patients, including acute respiratory distress syndrome and cryoshock. The cryoshock presented with multiorgan failure and disseminated intravascular coagulation, which was presented in three patients, contributing to death.

#### 3.1.4. Irreversible Electroporation

IRE is a novel non-thermal ablation technique. The mechanism of IRE is based on high-voltage electrical pulses that cause irreversible cellular membrane disruption, leading to cell death while keeping the underlying connective tissue scaffold intact. This technique keeps structures that are vulnerable to thermal injuries—such as blood vessels and bile ducts—intact. Because IRE does not use heat to eradicate the tumors, its efficacy is not impeded by the heat-sink effect on adjacent blood vessels [58]. IRE must be applied under general anesthesia, with complete muscle relaxation and ECG synchronization during IRE delivery [59]. A small case series study reported that luminal narrowing was noted in in 27.7 % (15/55) of bile ducts within a 1-cm radius of the ablation defect [31]. The primary efficacy of IRE ranges from 66 to 100% for hepatic tumors in proximity to major vascular or biliary structures [30,60,61,62,63,64]. The main limitation of IRE is that the ablation zone is created between two parallel electrodes spaced approximately 1–1.5 cm apart, which is relatively small compared to other techniques. The required precise placement of multiple electrodes in parallel with appropriate space is also challenging and time-consuming [65,66]. However, stereotactic guidance has been shown to be able to overcome these issues, significantly reducing procedure length and improving electrode placement accuracy [67]. For the evaluation of post-IRE ablation imaging, it has been reported that the imaging response may be inaccurate in the reflection of the histopathological appearance of the ablation zone, which limits the evaluation and efficacy of the treatment response [68,69].

Two retrospective studies found 2-year overall survival rates of up to 62% in CLM patients treated with IRE, and the 2-year progression-free survival was 18–40.5% [70,71]. Another study of 24 patients with unresectable CLM showed 3- and 5-year overall survival rates of 25% and 8.3%, respectively [62]. Recently, a phase II, single-arm clinical trial of unresectable CLM near critical structures reported a local control rate after 1 year following repeat IRE of 74%, and the overall complication rate was 40% [72]. Although this complication rate was substantially higher than most reports for the thermal ablation of CLM in less challenging locations, the authors inferred that most of the procedures having been performed simultaneously with surgery might be the cause of the high complication rate and not all of the reported adverse events were directly related to the IRE. Although the current evidence is encouraging, no current randomized controlled trials compare IRE with standard therapy. Moreover, the relatively higher rates of LTP with this technology might reflect the selection of tumors that are more challenging to treat due to their proximity to major bile ducts and vascular structures. Further randomized controlled trials comparing IRE with other standard treatments are warranted.

### 3.2. Imaging Guidance Techniques

Ultrasound, computed tomography (CT), magnetic resonance imaging (MRI), and positron emission tomography (PET) are utilized for liver ablation imaging guidance. Each modality has its strengths and weaknesses. Imaging fusion techniques for combining multiple modalities have also been developed for treatment planning, tumor targeting, and ablation margin assessment.

Pre-ablation imaging plays a critical role in patient selection and treatment planning [73]. A baseline intravenous contrast-enhanced CT scan of the chest, abdomen, and pelvis is commonly used in the workup of patients considered for ablation [74]. A whole-body fluorodeoxyglucose (FDG) PET/CT scan can provide additional information for better quantification of liver and extrahepatic metastases, and may change the management [75]. MRI is the most accurate imaging for the detection and characterization of hepatic metastases, especially with the hepatocyte-specific MRI contrast agent; it has a high sensitivity for the detection of smaller tumors that may not be easily detected by CT and PET [76]. The NCCN guidelines recommend that intravenous contrast-enhanced CT is the minimal requirement if liver-directed treatment is contemplated, and a hepatic MRI with intravenous extracellular or hepatobiliary gadolinium-based contrast agents is preferred over CT to assess the exact number and distribution of metastases [77].

#### 3.2.1. Computed Tomography

The advantages of CT are wide availability and decreased operator dependence. The intravenous contrast agent can be administered at the time of applicator placement to better localize the lesions. Using CT fluoroscopy can provide a nearly real-time visualization of applicator placement. An immediate post-ablation contrast-enhanced CT can be used to provide a rapid evaluation of ablation margins and residual tumors. The disadvantages of CT guidance include the ionizing radiation and the limitations of guidance planes.

#### 3.2.2. Magnetic Resonance Imaging

The advantages of MRI guidance include non-ionizing radiation, higher tissue contrast resolution, and multiparametric imaging. This allows for radiation-free near-real-time imaging during applicator placement [78]. Additionally, MRI is the only modality with well-validated techniques for near-real-time temperature monitoring during the ablation, which is useful to delineate the ablation zone [79]. The limitations of MRI guidance are the complexity of the procedure due to the use of only MR-compatible devices, limited availability, and relatively high cost.

#### 3.2.3. Ultrasound

Ultrasound can provide real-time monitoring of applicator placement without ionizing radiation. Although it is occasionally difficult to delineate the lesions due to its limited sensitivity and operator-dependent nature, using an intravenous contrast can improve the sensitivity, similarly to the contrast-enhanced CT [80,81]. A post-ablation contrast-enhanced ultrasound can provide an immediate evaluation of residual tumors and guidance for supplementary ablation [80]. One of the limitations of using ultrasound is that the gas bubbles generated during the RFA or MWA can obscure the visualization of the applicator and lesions.

#### 3.2.4. PET

The advantage of PET in ablation guidance is it can offer metabolic information during the procedure. Several studies have reported that PET can identify LTP following ablation earlier than intravenous contrast-enhanced CT before morphological changes, and PET/CT is currently recommended for the assessment and follow-up of patients with CLMs undergoing ablation [82,83,84]. However, there are challenges in the registration of images due to the morphological distortion after the ablation. Additionally, the FDG activity of tumors is not dissipated by ablation, and the ablation-related inflammatory changes can lead to difficulty in the assessment of residual tumors [85]. A split-dose technique for FDG PET/CT guidance has been developed to overcome these limitations [86]. The main concept of this technique is that a smaller first dose of FDG before the ablation will be significantly decayed by the time the second, larger dose is administered, allowing for the detection of FDG activity within any residual viable tumor. Another technique using intraprocedural nitrogen 13 ammonia perfusion PET has been developed to assess the ablation margins [87].

### 3.3. Stereotactic and Robotic Guidance

Stereotactic and robotic guidance systems have been used to facilitate the placement of the ablation applicator in real time [88,89], enhance the target visibility through image fusion [90], and improve the operator’s spatial orientation. Stereotactic guidance can be performed with electromagnetic or optical tracking systems. The instruments are guided either freehand, using an aiming device, or with the assistance of a robotic arm [91]. The most common imaging modalities used are three-dimensional (3D) CT and ultrasound, with developments towards the coupling of fluoroscopy and 3D imaging from C-arm cone-beam CT [92,93,94]. There is evidence from randomized trials showing that stereotactic or robotic guidance improves the accuracy of needle placement compared to conventional image guidance—especially when using off-plane trajectories [95,96]. In addition, it has been shown that the radiation dose in CT-guided ablation could be reduced using such devices [97,98].

## 4. Factors Affecting Ablation Outcomes

The increasing knowledge of factors affecting oncological outcomes after ablation has allowed CLM to be treated by ablation, with curative potential. Local tumor control of the ablated tumor is the main goal for effective ablation. Although ablation has a favorable curative potential for CLM, it has been associated with a higher risk of local recurrence when compared to surgical resection [14,99]. The desirable low LTP rates are associated with local disease control and disease-free survival, which might eventually affect overall survival.

### 4.1. Tumor Factors

Studies of LTP and survival show an advantage for small tumor size [15,37]. A tumor size up to 3 cm is an independent predictor of overall and LTP-free survival, and provides similar oncological outcomes to resection [15,16,17,100]. In a study of 233 CLM patients treated with RFA, tumors ≤ 3 cm in size had better LTP rates (44 vs. 78%; *p* < 0.001) and median overall survival (41 vs. 25 months; *p* = 0.005) [15]. Similar results were found in another study of 210 patients with CLM treated with MWA. Lower recurrence rates (47.9 vs. 66.2%; *p* < 0.001) and longer median overall survival (48.3 vs. 25.6 months; *p* < 0.001) were shown in patients with tumor size ≤ 3 cm [101]. For tumors of 3–5 cm in size, ablation can be considered with multiple overlaps to achieve complete ablation. Thermal ablation is generally not recommended for a curative intent to treat tumors larger than 5 cm because of the high local recurrence rate [7,19]. However, ablation of CLMs up to 5 cm in size can be performed with adequate planning and monitoring in selected cases, with acceptable outcomes [44,89].

The location of the tumor also affects local tumor control and complications. Ablation of tumors adjacent to central bile ducts using thermal ablation techniques is associated with increased risk of bile duct injury and subsequent complications, such as cholangitis or liver abscess [26]. The risk of tumor recurrence is increased in thermal ablation close to a large vessel (the heat-sink phenomenon), and warrants intensive treatment strategies [20,27,28]. In such circumstances, the non-thermal ablation technique IRE has a role in the treatment of tumors abutting central bile ducts and vessels [33,102].

### 4.2. Patient Factors

**Figure 1:**
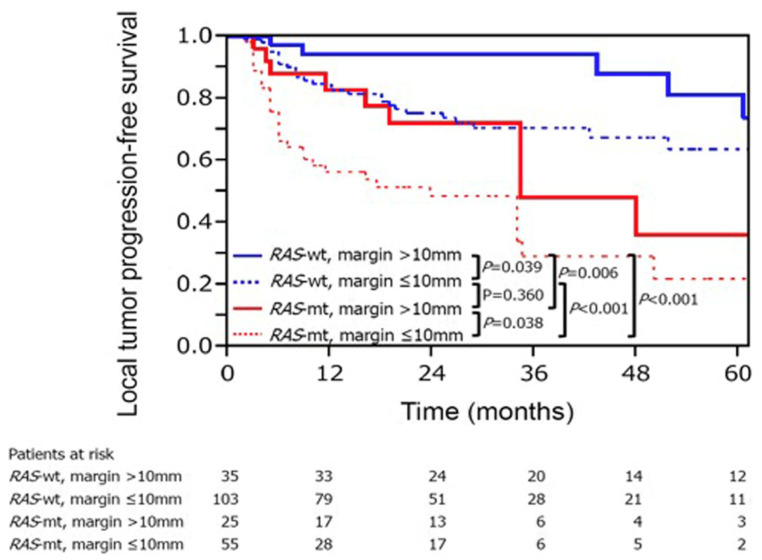
The *RAS* mutant status and the ablation margin are two important factors of LTP-free survival in CLM treatment with ablation. Reproduced with permission from Marco Calandri et al., European Radiology; published by Springer, 2018 [103].

Mutation of the *RAS* gene family (*KRAS*, *NRAS* and *HRAS*) is one of the relevant prognostic biomarkers for locoregional CLM treatment [103,104,105,106]. Mutations in the *RAS* gene family are present in up to 40% of patients with colorectal cancers [107]. Generally, patients with *RAS*-mutated CLM have worse survival than those with wild-type *RAS* [108,109]. Various studies have disclosed that patients with mutant *RAS* have earlier LTP and worse overall survival [103,105,106]. One study analyzed 92 patients with 137 ablated CLMs, and reported that those with mutant *RAS* had worse 3-year LTP-free survival than those with wild-type *RAS* (35 vs. 71%; *p* < 0.001) [104]. Additionally, *RAS* mutation was associated with earlier LTP in the ablated CLMs [104]. A two-institutional analysis of 136 patients with 218 ablated CLMs showed that achieving minimal ablation >10 mm can significantly improve the LTP among patients with mutant *RAS*. The 3-year LTP-free survival rates of mutant *RAS* CLM were 29% for ablation margins > 10 mm and 48% for ≤10 mm (*p* = 0.038) [103] (Figure 1).

Another study reported that the risk of LTP was 15.6-fold in mutant *RAS* with an ablation margin of 1–5 mm compared with wild-type *RAS* with an ablation margin of ≥6 mm [105]. Additionally, *KRAS* mutation was an independent predictor of shorter overall survival, time to the new liver, and peritoneal metastases. According to these studies, a minimal ablation margin of >10 mm should be acquired to achieve acceptable oncological outcomes, especially for mutant *RAS* tumors.

Beyond *RAS* alteration, other genetic alterations have prognostic utility in the treatment of CLM. Those include *BRAF*, *TP53*, *SMAD4*, and microsatellite instability [110]. Moreover, the double mutations of *TP53* with either *KRAS*, *NRAS*, or *BRAF* were associated with significantly worse survival compared with mutations in both gene groups alone in patients undergoing liver resection [111]. Coexisting mutations in *RAS*, *TP53*, and *SMAD4* were associated with significantly worse recurrence-free and overall survival than coexisting mutations in any two or one of these genes [112]. However, the evidence of these genetic profiles affecting the ablation outcomes is currently lacking.

The embryonic origin of the primary tumor can impact the oncological outcomes of patients with CLM treated by ablation. It has been reported that patients with primary tumors originating from the midgut region had worse survival when compared to patients with hindgut origin colorectal cancer [113,114]. In a study of 74 patients undergoing percutaneous thermal ablation, the 3-year recurrence-free survival and overall survival rates were 24% and 40%, respectively, for hindgut origin, and 5.6% and 8.3%, respectively, for midgut origin [113]. In this study, the patients with midgut-origin tumors had less local therapy in case of metastatic recurrence, inferring that the recurrence of midgut-origin tumors was more aggressive.

Moreover, prior history of liver resection was associated with local tumor control and survival after ablation of CLM. It has been reported that 3-year LTP-free survival (73 vs. 34%; *p* < 0.001), recurrence-free survival (23 vs. 9.1%; *p* = 0.026), and overall survival (78 vs. 48%; *p* = 0.003) were improved in patients with prior hepatectomy when compared to patients without history of prior hepatectomy for CLM [115]. The authors of this study speculated that the better oncological outcomes were attributed to the patient selection for initial hepatectomy, in which tumor characteristics are more favorable. According to these results, ablation can serve as an effective therapy for patients who present with newly developed CLM after liver resection.

Several patient and disease characteristics predicting clinical outcomes after thermal ablation could be used for patient stratification. An ablation clinical risk score adapted from surgical clinical risk score—including the nodal status of the primary tumor, the time interval from primary resection to CLM diagnosis, carcinoembryonic antigen level, number of tumors, and size of the largest tumor—is associated with local tumor control and overall survival [15,47,116].

For patients with extrahepatic disease, ablation can provide benefit to patients with limited and treatable extrahepatic diseases [34,117,118]. The patients with lung-only metastases had the highest median overall survival when compared with those with metastases at more than one site (35 months and 14 months, respectively) in a study of CLM treated with RFA [15]. The results were similar to a surgical report showing that patients who had only lung metastases had higher median overall survival compared with those with multiple metastatic sites (46 months and 15 months, respectively) in patients undergoing liver and extrahepatic disease resection [117].

### 4.3. Technique Factors

Several studies have shown the ablation margin as a key factor associated with local tumor control [15,25,37,119]. Minimal ablation margins larger than 5 mm in a 3D plane are associated with optimal local tumor control [15,37,119]. A panel of experts has recommended minimal ablation margins larger than 10 mm as a procedure goal for patients with CLM [14]. In a case series of 73 patients with 94 CLM tumors, the two-year LTP rates for tumors with 0-, 1–5-, 6–10-, or 11–15-mm minimal ablation margins were 74, 54, 26, and 20%, respectively (*p* = 0.011) [119]. In a recent report, the LTP rate for tumors with >10 mm ablation margins was 5%, compared to 90% and 60% for margins of 0 and 1–5 mm, respectively [15]. Nevertheless, achieving minimal ablation margins of >10 mm is a challenging task. From the published case series, the number of ablated CLM tumors with minimal ablation margins > 10 mm was less than 30% [15,103,119]. In contrast to liver resection, pathological determination of a margin-negative ablation cannot be made after most cases of image-guided ablation. However, ablation with negative biopsy findings of the margin and center of the ablation zone, and with ablation margins ≥ 5 mm, carries a cumulative incidence rate of LTP of 3% at 2 years, which is comparable to reported marginal recurrence after R0 resections for CLM [120,121]. Moreover, the negative biopsy findings were positively associated with ablation margins, indicating that achieving larger margins is comparable to complete resection. Nevertheless, the conventional method of measuring the ablation margins is by manual measurement with anatomic landmarks on post-ablation contrast-enhanced cross-sectional imaging [119]. This is limited by the misalignment of the liver due to the patient’s position and respiratory phases, tissue structural changes after ablation, and the image resolution. Some techniques using the non-rigid registration of pre- and post-ablation CT or MR imaging aim to solve the problem of misalignment and exploit perfusion PET or 3D software to facilitate the assessment of minimal ablation margins [81,87,122,123]; however, to date, no technique has been established as a reliable and objective source of intraprocedural information, warranting further investigation.

Accurate ablation applicator placement is a prerequisite during image-guided ablation. Stereotactic image-guidance systems have been proven to provide accurate 3D applicator placement to complete the tumor coverage [95,96]. This also allows for the placement of multiple applicators to cover the whole tumor with multiple overlapping ablation zones [89]. Some studies have reported that stereotactic image guidance can improve the primary efficacy of liver tumor ablation [98].

The precise tumor localization and conspicuity are important factors for successful image-guided ablation. It has been reported that transcatheter CT arterial portography and CT hepatic arteriography can improve the conspicuity of hepatic tumors during ablation [124]. A study of 108 patients with CLM submitted to 156 percutaneous thermal ablation procedures showed that CT hepatic arteriographic guidance was associated with significantly superior local tumor control compared with conventional CT fluoroscopic guidance. The 2-year LTP-free survival was 8.9% for CT hepatic arteriography and 32.8% for CT fluoroscopy (*p* < 0.001) [125].

The role of anesthetic techniques in ablation is critical, since they reduce the patient’s pain, anxiety, and movements during the procedure and facilitate the achievement of an adequate localization, resulting in a successful ablation [126]. Several anesthetic methods are used, such as general anesthesia, and sedation using fentanyl, midazolam, or propofol. Ideally, general anesthesia is preferred because of controllable respiration during the procedure, potentially allowing for more accurate ablation applicator placement and consequent ablation zones. For respiratory motion, high-frequency jet ventilation—a mechanical ventilation method—is applicable to decrease respiratory motion during liver ablation [127]. Nevertheless, sedation with propofol can provide equivalent oncological outcomes to general anesthesia. It has been reported that sedation with propofol and general anesthesia for percutaneous liver ablation are associated with better local tumor control than sedation with midazolam, providing LTP rates of 4.3% (4/94), 5.7% (2/35), and 45.2% (19/42), respectively (*p* < 0.001) [128].

## 5. Clinical Applications: Current Evidence

### 5.1. Patients Ineligible for Resection

Ablation has been used as a safe and effective treatment in patients with CLM initially ineligible for surgery [15,35,36,37] (Table 1). Patients who are ineligible for surgery comprise a significant portion of the present literature on the use RFA for CLM, with the 5-year overall survival rate ranging from 17% to 51% [15,36,129,130]. To date, only one randomized controlled trial has compared systemic chemotherapy with or without radiofrequency ablation, in which 119 patients with unresectable CLM were included [38,131]. In this phase II trial, patients with extrahepatic disease, more than 10 liver metastases, more than 50% liver involvement, and maximal tumor size of more than 4 cm were excluded. The study showed a significant improvement in terms of overall survival and progression-free survival for patients treated with combined treatment (RFA plus systemic chemotherapy). At a median follow-up of 9.7 years, the 8-year overall survival and progression-free survival rates were 35.9% and 22.3% for combined treatment, and 8.9% and 2.0% for systemic chemotherapy alone, respectively. It should be highlighted that almost half of the patients received RFA plus liver resection. Nevertheless, this trial seems to provide evidence that pursuing aggressive local treatment of CLM can prolong OS in patients with unresectable CLM.

For unresectable or unablatable CLM, neoadjuvant chemotherapy provides potential conversion to resectable or ablatable disease. Four-year survival of 45% has been reported by one study using RFA in a small group of 51 patients following neoadjuvant chemotherapy [137]. Nevertheless, neoadjuvant chemotherapy could result in the disappearance of liver metastases. A study of 325 CLM lesions identified by contrast-enhanced CT before neoadjuvant chemotherapy reported that only 183 lesions were identified after chemotherapy by CT, while 309 lesions were identified during intraoperative ultrasound [137]. The tumors that have disappeared on imaging can still be present upon pathological analysis, and are likely to recur on the follow-up imaging [138]. Therefore, complete radiological response is not correlated with complete pathological response, and further locoregional treatment is warranted. Furthermore, a recent meta-analysis reported that the evidence to support the routine use of neoadjuvant chemotherapy in repeat locoregional treatment is lacking, and a further randomized controlled study is under development [139]. Adjuvant chemotherapy could provide benefits for patients undergoing ablation compared to ablation alone. A study of 234 patients found improved median overall survival of 28 months versus 19 months for those who received adjuvant chemotherapy following RFA (*p* = 0.02) [140]. Another study of MWA reported a similar result, in which median overall survival was longer for patients with multiple lesions receiving adjuvant chemotherapy after ablation (46.7 vs. 25 months; *p* = 0.009) [101]. Nevertheless, such findings have not been validated in a prospective fashion.

### 5.2. Patients with Post-Resection Recurrence

Although repeat hepatectomy has been reported to be effective, not all of the patients are eligible for re-resection, due to limited liver reserve, unfavorable tumor location, and/or patient comorbidities. Ablation can be utilized as a salvage treatment for these patients, with recent series demonstrating outcomes similar to surgical resection [45,47,141,142]. Furthermore, ablation can be used as a “test-of-time” strategy for patients who had positive margins at liver resection, or for those recurring within 6 months of liver resection. It can offer additional local tumor control and prolong overall survival in a selected heavily pretreated population with limited therapeutic options [47]. A summary of studies evaluating liver ablation following hepatectomy is provided in Table 2.

### 5.3. Ablation in Combination with Surgical Resection

Patients with extensive distribution of CLMs pose a challenge for curative-intent local therapies. Liver resection combined with ablation has been applied with the goal of achieving cure and preserving the future liver remnant in these patients [39,42,145] (Table 3).

The combination of ablation with hepatectomy has been used to treat unresectable CLM. The benefits of adding ablation to hepatectomy include preserving the functional liver parenchyma and reducing the possibility of post-hepatectomy liver failure. A meta-analysis of seven studies from 2004 to 2017 compared hepatectomy to the combination of RFA and hepatectomy. The results showed that RFA in addition to hepatectomy for unresectable CLM resulted in comparable overall survival to hepatectomy alone. Moreover, the authors noted that among the eight studies published after 2012, seven showed similar OS when comparing ablation (±partial resection) to partial resection alone, which might indicate recent ablative technique improvements [151]. Regarding the combination of MWA with hepatectomy, a study of 53 patients showed no significant difference in overall survival between MWA plus hepatectomy and hepatectomy alone (median OS: 28 vs. 39 months; *p* = 0.43) [152]. A case series study reported a 5-year overall survival rate of 40.4% for MWA in addition to hepatectomy for unresectable CLM [39]. Conversely, a study compared two-stage hepatectomy to one-stage hepatectomy combined with RFA for bilobar CLM and found that two-stage hepatectomy improved the 5-year overall survival rate (35 vs. 24%; *p* = 0.01), with a lower incidence of postoperative hepatic insufficiency (6% vs. 28%, *p* < 0.0001) [147]. The authors suggested that surgeons may have inadvertently overablated the lesions in an attempt to reduce the local recurrence rate, but caused more unplanned damage to the future liver remnant.

In a case-matched study comparing patients treated with hepatectomy combined with RFA and those treated with hepatectomy alone, there were no significant differences in 5-year overall survival (57% vs. 61%, *p* = 0.573) or disease-free survival (19% vs. 17%, *p* = 0.865) [39]. Furthermore, a newly developed sequential treatment strategy—planned incomplete resection followed by postoperative percutaneous completion ablation for intentionally untreated lesions—has shown that this novel strategy may provide better local tumor control (5-year local tumor recurrence: 31.7 vs. 62.4%; *p* = 0.03) and lower complication rates when compared with intraoperative ablation, while showing no significant differences in 5-year overall survival (53.2 vs. 41.8%; *p* = 0.407) [42].

## 6. Conclusions and Future Perspective

The role of ablation in the management of CLM has significantly evolved in recent years. Ablation is a well-recognized local treatment option that can provide great local tumor control for small CLMs where adequate minimal ablation margins can be achieved. When combined with systemic chemotherapy, ablation can provide survival benefits for patients with unresectable CLM when compared to chemotherapy alone. Ablation also allows safe, cost-effective longitudinal sequential locoregional therapy with curative intent for recurrent CLM, which is common after initial CLM resection. Further understanding of the impact of tumor biology, the use of advanced imaging guidance for procedure planning and assessment, and the expected results of ongoing clinical trials will help to tailor its application as a local cure for patients with CLM.

## Figures and Tables

**Table 1:** Relevant published studies on survival following ablation of colorectal liver metastases initially ineligible for resection alone within 10 years.

Author/Year	Type of Study	Intervention	Approach	Number of Patients/Lesions	Mean/Median Tumor Size (cm)	Mean/Median Tumor Number	Median Follow-Up Period in Months	3-y OS (%)	4-y OS (%)	5-y OS (%)
Tinguely/2020 [132]	Retrospective	MWA	Percutaneous or surgical	82	3 *	n/a	25.2	69.1	n/a	n/a
Thai Doan/2020 [133]	Retrospective	RFA	Percutaneous	61	n/a	2.7	24	44.5	n/a	38.2
Cornelis/2020 [70]	Retrospective	IRE	Percutaneous	25	n/a	n/a	25	26.8	n/a	n/a
Schicho/2019 [62]	Retrospective	IRE	Percutaneous	24	2	2	26.5	25	n/a	8.3
Ruers/2017 [38]	Prospective	RFA ± resection	Percutaneous or surgical	60 ^†^	4 *	4	116.4	56.9	n/a	43.1
Engstrand/2014 [134]	Retrospective	MWA	Surgical	20	2.7	9	25	n/a	41	n/a
Evrard/2012 [135]	Prospective	RFA ± resection	Surgical	52	1	5	34.8	n/a	n/a	43
Kim/ 2011 [129]	Retrospective	RFA	Percutaneous or surgical	177	2.1	1.6	41.2	n/a	n/a	51
Van Tilborg/2011 [136]	Retrospective	RFA	Percutaneous or surgical	100	2.4	1.9	29	77	n/a	36

Abbreviations—IRE: irreversible electroporation; MWA: microwave ablation; n/a: not available; OS: overall survival; RFA: radiofrequency ablation. *: Maximal diameter. ^†^: combined modality arm (systemic treatment plus aggressive local treatment by radiofrequency ablation ± resection).

**Table 2:** Relevant studies on survival following ablation of recurrent colorectal liver metastases after hepatectomy within 10 years.

Author/Year	Type of Study	Number of Patients/Lesions	Approach of Ablation	Mean/Median Tumor Size (cm)	Mean/Median Tumor Number	Median Follow-Up Period in Months	Local Tumor Progression Rate (%)	Liver Limited Recurrence (%)	Repeat Local Treatment for Liver Limited Recurrence (%)	3-y OS (%)	5-y OS (%)
Fan/2020 [143]	Retrospective	144/258	Percutaneous RFA	2.6	5.1	28.6	7	79.2 *	n/a	n/a	27.1
Zimmermann/2020 [43]	Retrospective	23/29	Percutaneous RFA	n/a	n/a	26	n/a	74	n/a	57	24
Schullian/2020 [44]	Retrospective	64/217	Percutaneous RFA ^§^	2.7	2	21	11.5	48.4 *	48.4	46.2	34.8
Mao/2019 [144]	Retrospective	61/114	Percutaneous RFA ^#^	2.7	2	28.9	16.7	54.1	52	n/a	33
Odisio/2018 [115]	Retrospective	49/59	Percutaneous RFA, MWA, and Cryoablation	n/a	n/a	28	5.1	n/a	n/a	78	n/a
Dupré/2017 [45]	Retrospective	33/n/a	Open or percutaneous RFA, MWA, and IRE	2	2	36.2	n/a	54.5	88.9	30.4	n/a
Sofocleous/2011 [47]	Retrospective	56/71	Percutaneous RFA	1.9	1.3	22	50.7	n/a	47.2	41	n/a

Abbreviations—n/a: not available; OS: overall survival, *: Intrahepatic distant metastases; ^§^: stereotactic radiofrequency ablation. Retreat with RFA only. ^#^: Resectable CLM.

**Table 3:** Relevant studies published in the last 10 years on ablation + surgery versus surgery alone for colorectal liver metastases.

Author/Year	Type of Study	Modality	Number of Patients	Mean/Median Tumor Size (cm)	Mean/Median Tumor Number	Median Follow-Up Period (Months)	Liver Limited Recurrence Rate (%)	Repeat Local Treatment for Liver Limited Recurrence (%)	3-y DFS (%)	5-y DFS (%)	3-y OS (%)	5-y OS (%)
van Amerongen/2019 [146]	Retrospective	RFA + resection	18	2.7	3	28	n/a	n/a	0	n/a	43	n/a
		Resection	63	3.2	1	28	n/a	n/a	16	n/a	72	n/a
Mizuno/2018 ^†^ [147]	Retrospective	1S ± RFA	101	4	5	39	n/a	n/a	n/a	n/a	n/a	24
		2S	126	3.4	7	39	n/a	n/a	n/a	n/a	n/a	35
Hof/2018 [148]	Retrospective	RFA ± resection ^#^	35	1.9	n/a	36.1	n/a	n/a	n/a	39.1	n/a	49.2
		Resection	35	2.2	n/a	36.1	n/a	n/a	n/a	30.1	n/a	56.3
Imai/2017 [40]	Retrospective	RFA + resection	31	1.4 (RFA) 3 (resection)	2 (RFA) 5 (resection)	35.6	58	59	n/a	19	n/a	57
		Resection	93	3.3	5	35.6	47	51.1	n/a	17.9	n/a	61
Sasaki/2016 [149]	Retrospective	RFA + resection	86	2.2	5	30.9	n/a	n/a	n/a	n/a	52.6	37.2
		Resection	399	2.5	2	30.9	n/a	n/a	n/a	n/a	73.8	58.7
Faitot/2014 * [145]	Retrospective	1S ± RFA	78	n/a	9.7	47	n/a	34	11	11	52	35
		2S	78	n/a	9.6	39	n/a	28	12	8	49	29
Eltawil/2014 [150]	Retrospective	RFA + resection	24	3.3	3	36	50	n/a	13	n/a	66	n/a
		Resection	150	2.7	1	35	25	n/a	29	n/a	61	n/a
Kim/2011 [129]	Retrospective	RFA + resection	27	2.1	3.1	21.6	n/a	n/a	n/a	18.4	n/a	22.9
		Resection	95	2.6	1.5	21.6	n/a	n/a	n/a	16.2	n/a	34.6
Okuno 2020 [42]	Retrospective	RFA/MWA + resection	92 ^a^ 23 ^b^	1.1 ^a^ 1 ^b^	4	39.6	n/a	n/a	n/a	n/a	n/a	42 ^a^ 53 ^b^

Abbreviations—DFS: disease-free survival; n/a: not available; OS: overall survival; 1S: one-stage hepatectomy; 2S: two-stage hepatectomy; *: In this study, patients were divided into one-stage and two-stage hepatectomy. The RFA was carried out on 92% of the one-stage group and 8% of the two-stage group; ^†^: In this study, patients were divided into one-stage and two-stage hepatectomy. The RFA was carried out on 71% of the one-stage group and none of the two-stage group. ^#^: In these 35 patients, 9 patients underwent RFA only; ^a^: intraoperative ablation; ^b^: planned incomplete resection and postoperative percutaneous completion ablation under cross-sectional imaging guidance for intentionally untreated tumors.
